# Safety Evaluation of Fermented and Nonfermented *Moringa oleifera* Seeds in Healthy Albino Rats: Biochemical, Haematological, and Histological Studies

**DOI:** 10.1155/ijfo/2694100

**Published:** 2025-03-24

**Authors:** Foluso Olutope Adetuyi, Emmanuel Sina Akintimehin, Kayode Olayele Karigidi, Abimbola Oluwatayo Orisawayi

**Affiliations:** ^1^Department of Chemical Sciences, Olusegun Agagu University of Science and Technology, Okitipupa, Ondo State, Nigeria; ^2^Composites and Advanced Materials Centre, Cranfield University, Cranfield, Bedfordshire, UK; ^3^Department of Mechanical Engineering, Olusegun Agagu University of Science and Technology, Okitipupa, Ondo State, Nigeria

**Keywords:** biochemical analysis, fermentation, histopathology, *Moringa oleifera* seed, oxidative stress, supplementation

## Abstract

Fermentation preserves and enhances food properties, but consuming locally fermented foods can cause health issues like flatulence, gastrointestinal disorders, kidney stones, and sometimes death. This study evaluated the biochemical, haematological, and histological effects of supplementing diets with fermented *Moringa oleifera* seed (FMS) and nonfermented *Moringa oleifera* seed (NFMS) in healthy albino rats. Male rats were fed diets containing 10%, 20%, and 30% FMS and NFMS for 14 days. No significant changes were observed in body weight or organ/body weight ratios. However, platelet count increased significantly (*p* < 0.05) at higher supplementation levels, suggesting enhanced haemostatic activity. While haematological parameters remained stable, NFMS at 20% and 30% increased urea and creatinine levels, indicating potential renal stress. Histological analysis showed mild alterations at higher supplementation levels, more pronounced in NFMS-fed rats. Fermentation mitigated antinutrient effects, enhancing safety. FMS and NFMS are safe up to 20% inclusion, with potential applications in human nutrition and functional food development.

## 1. Introduction

There is a growing trend in the consumption of both fermented and nonfermented plant-based formulations as alternative medicines, driven by the strong belief that these products are safe and free from toxic side effects when used as alternatives to conventional pharmaceutical drugs [1, 2].

One important example is *Moringa oleifera* (*M. oleifera*), known as a unique plant whose seeds are cheaply available and valued for their medicinal properties and versatility [3]. *M. oleifera* seeds are valued for their nutritional and medicinal properties, containing essential vitamins, minerals, fats, and bioactive phytochemicals [4]. These bioactive compounds, including glucosinolates, isothiocyanates, alkaloids, tannins, flavonoids, and glycosides, contribute to the plant's therapeutic applications in health promotion, disease prevention, and environmental sustainability [5, 6]. In traditional practices in Asia and Africa, *M. oleifera* seeds are conventionally used in some parts for water treatment applications [6, 7]. This was also supported by a few studies that have been conducted in some parts of Europe and have shown the innovative combination of *M. oleifera* seeds with alginate biodegradable polymer as a potential biosorbent for water treatment application [3]. In addition to the purification function, the residue obtained from the seed after defatting the seed is often used as an adjuvant or fertilizer for processing animal feed in some regions [8].

The *M. oleifera* seed was also reported to contain important phenolic compounds such as gallic acid, ellagic acid, and kaempferol, with levels varying depending on the region where the seed is grown [10, 11]. The presence of these phytoconstituents in *M. oleifera* seed has been reported to significantly contribute to its nutritional values, protection against oxidative-induced DNA damage, protection against several inflammatory pathological conditions, antihypertensive activity, and antimicrobial activity [13].

Fermentation is one of the ancient, simple, sustainable, and low-cost biotechnology techniques performed on fruits, vegetables, and other food products to retain the vital properties that enable satisfactory organoleptic features and enhanced microbial stability, thereby extending the shelf life of the food products [14]. This technology sometimes not only extends the shelf life of food products but also inhibits the growth of hybrid bacteria, resulting in better fermentation flavor, nutritional value, and greater product variety. This, therefore, improves the taste and palatability characteristics and enhances the nutritional, detoxification, and functional (bioactive constituents) properties of fermented food [15].

Studies showed that during fermentation, lactic acid bacteria (LAB), along with various bacteria, yeast, and filamentous fungi, play major roles. A recent report on the impact of fermentation on *M. oleifera* seeds revealed improvement in proximate composition, the nutrient profile (such as essential amino acids and polyunsaturated fatty acids), and a reduction in antinutrient factors [16]. Aside from loads of microorganisms (serving as probiotics) with huge health benefits in fermented foods, enterotoxigenic and enterohemorrhagic pathogens have also been detected in fermented foods and consequent production of toxic metabolites [17, 18].

Despite the extensive use of *M. oleifera* in traditional medicine, food, and industrial applications, there are limited scientific data on the safety of fermented *M. oleifera* seed (FMS) and nonfermented *M. oleifera* seed (NFMS) for consumption [6, 18]. While our studies aimed at exploring the nutritional and medicinal benefits of NFMSs, there remains little experimental evidence on how fermentation affects its biochemical safety, haematological responses, and histopathological impacts in vivo. To the best of our knowledge, this work pioneers the evaluations of the safety between the FMS and NFMS using in vivo biochemical, haematological, and histological assessments in male albino rats. This study explores whether the fermentation mitigates the potentially toxic effects of the *M. oleifera* seed supplementation, providing insights into safe dietary inclusion levels and potential functional food applications. The key findings from this study could provide scientific evidence for the determination of how fermentation enhances the safety and bioavailability of *M. oleifera* for dietary applications in humans.

## 2. Materials and Methods

### 2.1. Plant Material and Preparation

Dry *M. oleifera* pods were collected from a farm product trusted supplier in Okitipupa, Ondo state, Nigeria. The seed was authenticated at the herbarium unit of the Biological Sciences Department, Olusegun Agagu University of Science and Technology (OAUSTECH), Nigeria. The dry pods were break-opened to remove the seed kernel with husk. The *M. oleifera* seed kernel was dehulled to remove the seed, which was then air-dried for 2 weeks and divided into two parts, labelled FMS and NFMS.

### 2.2. *M. oleifera* Seed Fermentation and Commercial Feed Fortification

Each portion (1.5 kg) of FMS and NFMS was separately blended with distilled water and drained with a muslin cloth to remove the water. NFMS was immediately sundried for 1 week while the FMS-labelled sample was transferred into the sac and kept at room temperature for 72 h for fermentation to take place and thereafter sundried for 1 week. After sun-drying, both samples were blended separately again to a powder form for further study. Fortification of commercial rat feed with *M. oleifera* seed followed the method described by Nwonuma et al. [19]. The rat feed was fortified by substituting 10% *M. oleifera* seed (10 g FMS/NFMS + 90 g of normal feed), 20% substituted feed (20 g FMS/NFMS + 80 g of normal feed), and 30% substituted feed (30 g FMS/NFMS + 70 g of normal feed). The supplementation levels of 10%, 20%, and 30% were chosen based on previous studies evaluating the safety and efficacy of *M. oleifera* in animal models [20, 21]. Since too much *M. oleifera* seed can lead to digestive issues, kidney damage, and infertility because of its high content of alkaloids and antinutrients [22], the hepatoprotective potential of *M. oleifera* justifies its use in diet supplementation within the selected range.

### 2.3. Experimental Animal and Treatments

Thirty-five male albino rats (160–200 g) were used in our study. The rates were obtained from Ladoke Akintola University of Science and Technology (LAUTECH), Ogbomosho, Nigeria. Before the experiments, the rats were placed in a spacious room with good ventilation with free access to rat chow and water for 2 weeks. Subsequently, animals were randomly divided into four groups of five animals each. Animals in Group 1 were given normal rat feed and water, serving as a control. Animals in Groups 2, 3, and 4 were divided into FMS and NFMS groups and given normal rat feed fortified with 10%, 20%, and 30% of FMS and NFMS. Animals were fed for 14 days, and rats' weights were recorded at intervallic (5 days) intervals. All animal studies were approved by the research ethics committee of OAUSTECH (Approval Number: OAUSTECH/ETHC-BCH/2023/011) and conducted under the ethical framework of the Federal Ministry of Health (Nigeria), National Health Research Ethics Committee (NHREC), Nigeria, regulations for research involving animals.

### 2.4. Experimental Termination and Sample Collection

At the end of the experiment, animals were allowed to fast overnight for 12–16 h before sacrifice; this fasting period is recommended to stabilize metabolic parameters and ensure accurate biochemical measurements. During the fasting period, animals were provided with water ad libitum to prevent dehydration [20]. After this, blood samples were carefully withdrawn via ocular puncture using a heparinized capillary tube and dispensed in appropriate sample bottles (EDTA for haematology and lithium heparin for other blood chemistry). Blood samples for blood chemistry were centrifuged for 10 min at 4000 × g to obtain the plasma. The liver and kidney were removed immediately, rinsed with normal saline (0.9% NaCl), and weighed. Excised tissues were homogenized with physiological phosphate-buffered saline (PBS) and centrifuged at 5000 × g for 15 min to obtain supernatant for determination of oxidative stress markers. Portions of excised tissues were fixed in 10% formalin for histopathology.

### 2.5. Biochemical Analysis of Blood Sample

#### 2.5.1. Haematological Analysis

The haematological parameters, which include the red blood cell (RBC) count, white blood cell (WBC) count and differentials, total hemoglobin (TH), platelet (PLT) count, haematocrit (HCT), and mean corpuscular volume (MCV), were analyzed in whole blood obtained from the rats using an automated haematology analyzer (Model: URIT-2900 Plus 3 Differential, URIT Medical Electronic Group Co. Ltd., Guilin, China) [24].

#### 2.5.2. Blood Chemistry Analysis

Blood glucose concentration, total protein, albumin, bilirubin, lipid profiling, and liver and kidney function indices were performed using diagnostic kits purchased from Fortress Diagnostics, Antrim, UK. Parameters for lipid profiling were estimated using kits from Randox Diagnostic Reagents (Randox Laboratories Ltd., UK). Biochemical parameters in blood plasma were quantified using a T70 PG UV-visible spectrophotometer (PG Instruments Ltd., Lutterworth, UK). The spectrophotometer, with wavelength ranges from 190 to 1100 nm, high accuracy (±0.3 nm), and repeatability (±0.2 nm), was employed to ensure precise and reliable measurements. The detailed biochemical assays measured included blood glucose, measured using the glucose oxidase-peroxidase (GOD-POD) method at 505 nm; total protein, determined by the biuret method at 540 nm; albumin assay using the bromocresol green method at 630 nm; and bilirubin, quantified specifically using the diazo method at 540 nm. Lipid profiling, cholesterol, and triglycerides were measured at 500 and 540 nm, respectively. Finally, liver and kidney function indices were also determined. The UV-Win software recommended by the manufacturer with the T70 PG UV-visible spectrophotometer was used for detailed acquisition and comprehensive analysis, ensuring accuracy and reliability in the quantification of these biochemical parameters [24].

### 2.6. Total Protein Determination in Tissues

The method of Ma et al. [22] was used for the determination of total protein concentration in tissue homogenates.

### 2.7. Oxidative Stress Markers in Tissues

Indices of oxidative stress markers that were analyzed are superoxide dismutase (SOD), catalase, reduced glutathione, and lipid peroxidation (LPO), which were measured using the reported method with slight modifications to suit our experiment [23, 24].

### 2.8. Histological Analysis

The histological analysis was done considering several studies from previous literature. Organs that had been kept in formalin were embedded in paraffin. A microtome cutter was used to slice embedded tissues with a thickness of 5 mm. The samples were stained with haematoxylin and eosin, and tissue sections were examined under an inverted microscope at 400× to observe histological changes in the organs [24].

### 2.9. Statistical Analysis

Analysis of variance (ANOVA) was used to statistically analyze the data generated, expressed as mean ± standard error of the mean (SEM). Statistical significance of mean difference was tested using least square difference (LSD) post hoc test at *p* < 0.05.

## 3. Results and Discussion

### 3.1. Biochemical Effects of *M. oleifera* Seed on Body and Organ Weight Ratios in Rats

**Figure 1:**
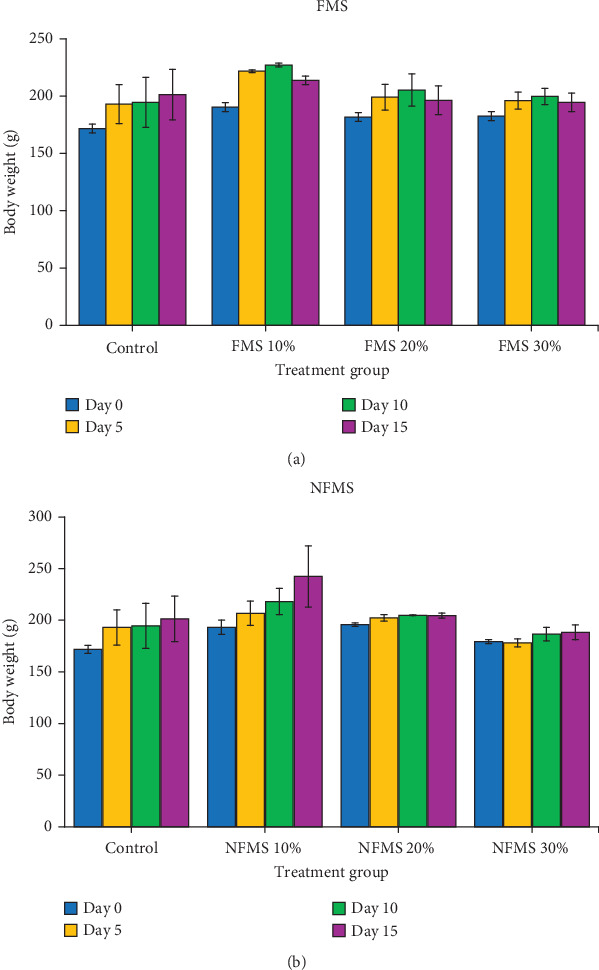
Periodic body weight of rat: (a) FMS 10%: 10 g fermented *M*.*oleifera* seed + 90 g of normal feed, FMS 20%: 20 g fermented *M*.*oleifera* seed + 80 g of normal feed, and FMS 30%: 30 g fermented *M*.*oleifera* seed + 70 g of normal feed and (b) NFMS 10%: 10 g nonfermented *M*.*oleifera* seed + 90 g of normal feed, NFMS 20%: 20 g nonfermented *M*.*oleifera* seed + 80 g of normal feed, NFMS 30%: 30 g nonfermented *M*.*oleifera* seed + 70 g of normal feed.

**Figure 2:**
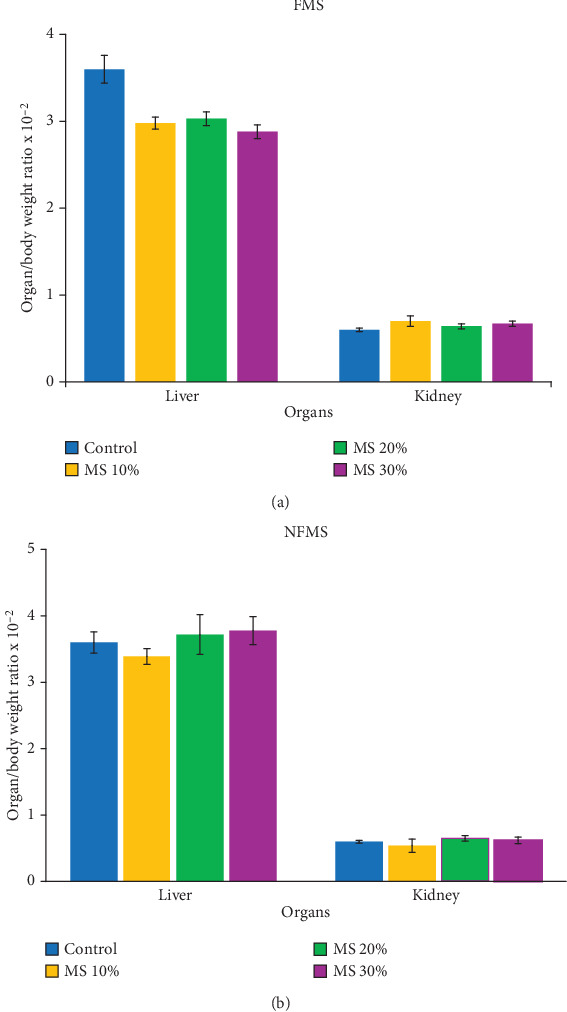
Organ/body weight ratio of rats administered to *M. oleifera* seed: (a) FMS 10%: 10 g fermented *M*.*oleifera* seed + 90 g of normal feed, FMS 20%: 20 g fermented *M*.*oleifera* seed + 80 g of normal feed, and FMS 30%: 30 g fermented *M*.*oleifera* seed + 70 g of normal feed and (b) NFMS 10%: 10 g nonfermented *M*.oleifera seed + 90 g of normal feed, NFMS 20%: 20 g nonfermented *M*.*oleifera* seed + 80 g of normal feed, and NFMS 30%: 30 g nonfermented *M*.*oleifera* seed + 70 g of normal feed.

The periodic body weight of rats till the termination of the experiment is shown in both Figure 1a,b, while the organ/body weight ratio of rats administered to *M. oleifera* seed is shown in Figure 2a,b, respectively. The result showed no significant difference in the body weight of rats fed with supplemented feed containing FMS and NFMS compared to untreated groups. Similarly, the organ/weight ratio of the liver and kidney of treated rats statistically remains unchanged relative to the control over 14 days. These observations could signify that NFMS and FMS are palatable for consumption and do not suppress appetite or feeding patterns. In consonance with previous studies, the stable body weight and organs relative to the untreated groups could similarly portray the nontoxic feature of the seed [25].

### 3.2. Haematology Parameters and Blood Chemistry Parameters

#### 3.2.1. Haematological Analysis Findings and Results

Haematological indices remain vital toxicological tools for assessing pathophysiological levels or clearance of toxicants in the circulatory system of men and experimental rats [30, 31]. The result in Table 1 showed the haematology parameter results of albino rats that were fed with FMS and NFMS. It was observed that there was no significant difference in WBC, MID, RBC, Hb, and MCV of rats that were given both FMS and NFMS. The nonsignificant changes that were observed in the RBC, Hb, WBC, MID, and MCV of rats that were fed with FMS and NFMS could indicate unaltered blood formation in the bone marrow and maintenance of normal physiological blood functions. The lymphocyte (LYM) decreased (*p* < 0.05) significantly in animals that received NFMS, while the values remained unchanged in animals that received FMS. HCT significantly increased in animals that received 20% and 30% of FMS, while NFMS supplementation caused no significant changes. Varying substitutes of NFMS and 30% FMS caused a significant (*p* < 0.05) increase in PLT count. This suggests enhanced haemostatic activity and thrombopoiesis, rather than a toxicological concern. PLTs play a crucial role in blood clotting, and this increase could indicate an adaptive response rather than an adverse effect. The observed significant (*p* < 0.05) decrease in LYMs of animals that received NFMS might not necessarily point to disturbance of the body's immune system since WBC and other differentials demonstrated undistinguished changes relative to control. However, alterations in the level of LYMs may have been linked to studies reported in association with low infection resistance since it serves as the main effector cell of the immune system [32]. A possible cause of this observation may also be due to the reduction due to the presence of some bioactive agents in NFMS samples that interfere with LYM activity. The significant rise in HCT of rats that were fed with 20% and 30% of FMS could be linked to activities of essential nutrients and bioactive compounds that help to prevent anaemic conditions [33, 34].

#### 3.2.2. Blood Chemistry Analysis Findings and Results

The results obtained for the blood chemistry of animals that were fed with substituted *M. oleifera* feed are presented in Table 2. The result shows that only 30% of NFMS caused a significant decrease in glucose concentration, while other groups were indistinguishable in comparison with the control. Total protein, ALT, AST, and ALP were statistically not significant to control, while there were substantial differences in GGT activity across the tested doses. Except for a group that received 30% NFMS that demonstrated a significant (*p* < 0.05) increase in bilirubin level, other treated groups showed no significant difference compared to the control. A 20% and 30% NFMS caused a significant (*p* < 0.05) increase in urea and creatinine levels, indicating potential renal stress. This may be attributed to the presence of antinutritional factors such as phytates and oxalates in NFMS [29]. The findings suggest that higher consumption of NFMS may require caution due to its potential renal burden, whereas fermentation appears to mitigate these effects. Similar findings have been reported in studies on plant-based extracts, where extraction and processing techniques influence bioactive safety and bioavailability [35], while uric acid was significantly reduced in animals that received only FMS. Further, animals fed with both FMS and NFMS showed a significant reduction in cholesterol and LDL concentration. Statistically, FMS did not alter triglyceride concentration, while NFMS caused a noticeable decrease in triglyceride concentration compared to control. Both FMS and NFMS did not alter the level of plasma high-density lipoprotein (HDL). In corroboration with previous findings on the effects of *M. oleifera* seed in diabetic-induced animal models, this study has demonstrated that the fermentation of *M. oleifera* seed does not alter the blood-lowering potential of the seed [36]. Determination of total plasma protein revealed that both FMS and NFMS confer no detrimental effect on protein synthetic function. Significant elevation of GGT was observed in groups that were administered with 20% and 30% NFMS, while ALT, AST, and ALP activities were unperturbed; this observation could be suggestive of a nonseverance effect of both FMS and NFMS. Additionally, except for the 30% NFMS that significantly elevated bilirubin, other treated groups showed the absence of abnormalities in the metabolism of bilirubin. Possible factor(s) that could account for the obvious increase in bilirubin concentration include disorder of the liver, bile ducts, or an increased rate of heme destruction. However, it is interesting to note that indications of severe liver injury are frequently observed when the concentration of ALT, ALP, GGT, and bilirubin is two- to threefold higher than the reference concentration [37].

The kidneys play a vital role in the excretion of waste products and toxins such as urea, creatinine, and uric acid; the regulation of extracellular fluid volume; and electrolyte concentrations. The sharp increase in the concentration of any of these waste products occurs when there is an increased metabolic rate of proteins, muscle bulk damage to the kidney, or a malfunctioning nephron system [38]. Administration of 20% and 30% NFMS caused significant changes in kidney function. At the same time, FMS has no observed difference compared to the control. The elevated urea/creatinine ratio may provide a biochemical signature of increased muscle catabolism in rats fed a high-phytate diet [39]. The elevated urea and creatinine levels in NFMS-fed rats compared to FMS suggest that antinutritional factors such as phytates and oxalates may contribute to impaired kidney function by reducing mineral metabolism and increasing renal filtration load (Table 2). This is consistent with studies demonstrating that unprocessed plant-based bioactive can lead to nephrotoxicity due to excess retention of antinutritional factors [35]. Fermentation has been shown to degrade these compounds, thereby reducing their adverse effects on renal clearance and excretion pathways and creatinine in NFMS-fed rats compared to FMS. This might be linked to some amounts of certain minerals (calcium and phosphorus) and antinutrients (such as phytate) that were also reported in raw *M. oleifera* seed [40]. The decrease in the urea and creatinine in FMS when compared to NFMS could be due to the removal of phytate by fermentation because a high intake of phytate caused an increase in urea and creatinine leading to renal stress. Previous studies have implicated high dietary intake of calcium as one of the risk factors for the development of kidney stones and subsequent renal damage [41].

Uric acid, which is formed from the metabolism of purine, was significantly reduced in groups that were fed with FMS, while NFMS demonstrated no significant difference. The level of uric acid in serum is measured by the difference between purine consumption and uric acid production as well as uric acid removal by the kidney. The probable reason for the uric acid reduction may be linked to the degradation of purines by microorganisms (probiotics) during fermentation [42].

However, both FMS and NFMS demonstrated significant lipid-lowering effects, evident in the reduction of triglyceride, cholesterol, and LDL levels (Table 2). This aligns with previous findings that plant-derived bioactives, such as flavonoids and polyphenols in the study of the antidiabetic potential of *Senna siamea*, contribute to hypolipidemic effects through enzyme inhibition and metabolic regulation [43]. These effects are crucial for the management and prevention of atherosclerosis, a leading cause of cardiovascular disease (CVD). The antioxidant potential of phytochemicals in *M. oleifera* may also contribute to this hypolipidemic action by reducing LPO and improving lipid metabolism [44], which is a major cause of CVD. Lipid-lowering is crucial for the prevention of CVD because it reduces the risk of heart attacks, strokes, and other cardiovascular events. Lowering the LDL in the blood will reduce the buildup of plaque in arteries and improve overall cardiovascular health [45]. Both FMS and NFMS were able to perform these functions, probably because of their crude fibre content and phytate. Despite its adverse effects on dietary phytates in animal and human health, it can be beneficial because it inhibits digestive enzymes that hydrolyse lipids, proteins, and starch [46, 47].

### 3.3. Total Protein and Oxidative Stress Marker in Organs

Oxidative stress frequently occurs due to the overwhelming level of pro-oxidants against the antioxidant defence capacity of the cells [48]. The result of the total protein and oxidative stress markers in the liver is presented in Table 3, while the total protein and oxidative stress markers in the kidney are presented in Table 4. Oxidative stress markers are very important to assess the level of oxidative damage in the body. This allows for proper evaluation of the potential risk of diseases, showing disease progression and antioxidant interventions' effectiveness in oxidative stress [49]. It was observed in this study that the consumption of *M. oleifera*–fortified rat feed for 14 days sustained protein synthesis function in both the liver and kidney. *M. oleifera* seed is well known to be an excellent source of proteins and essential amino acids for promoting human nutrition and health [50, 51]. It is shown in this study that liver CAT significantly reduced in animals that received only NFMS, while FMS caused no significant difference in liver CAT activity. The liver CAT was significantly reduced in animals that received only NFMS, while FMS caused no significant difference in liver CAT activity. This could be due to the biological activities of bioactive compounds in the NFMS sample. Except for liver catalase, other antioxidant systems as well as MDA in the liver and kidney of animals that were fed FMS and NFMS remain unaltered compared to control. This observation could suggest that both FMS and NFMS might contain important constituents to compensate for assaults that could arise from oxidative stress. In the kidney, only animals that received 10% substituted *M. oleifera* seed (FMS and NFMS) demonstrated a significant (*p* < 0.05) decrease in catalase activities. At the same time, other treated groups showed no difference compared to the control. The level of GSH and SOD activities in both liver and kidney were not affected by FMS and NFMS administration. Supplementation of FMS and NFMS at 20% and 30% resulted in a significant decrease in liver MDA, while the varying percentage of both FMS and NFMS caused no significant changes in kidney MDA.

### 3.4. Histopathological Examination

**Figure 3:**
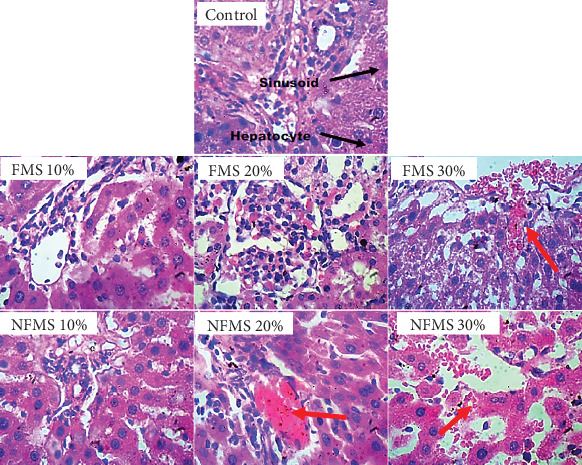
Panoramic and magnified views of a liver micromorphological section demonstrated by haematoxylin and eosin staining at high magnification (400×). The hepatocytes, sinusoids, and portal triad (hepatic vein, hepatic artery, and bile duct) are all visible across the various groups. FMS 10%, 10 g fermented *M*.*oleifera* seed + 90 g of normal feed; FMS 20%, 20 g fermented *M*.*oleifera* seed + 80 g of normal feed; FMS 30%, 30 g fermented *M*.*oleifera* seed + 70 g of normal feed; NFMS 10%, 10 g nonfermented *M*.*oleifera* seed + 90 g of normal feed; NFMS 20%, 20 g nonfermented *M*.*oleifera* seed + 80 g of normal feed; NFMS 30%, 30 g nonfermented *M*.*oleifera* seed + 70 g of normal feed.

**Figure 4:**
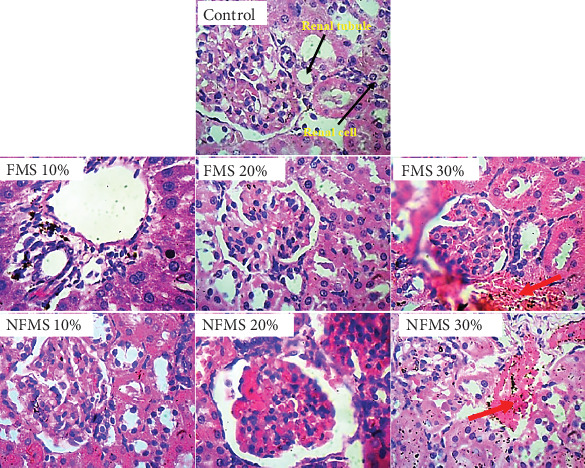
Panoramic and magnified views of kidney micromorphological section demonstrated by haematoxylin and eosin staining at high magnification (400×). The renal cortex, renal tubules, glomeruli, and mesangial cells as well as renal parenchymal cells and glomerular capsules are all visible across the various groups. FMS 10%, 10 g fermented *M*.*oleifera* seed + 90 g of normal feed; FMS 20%, 20 g fermented *M*.*oleifera* seed + 80 g of normal feed; FMS 30%, 30 g fermented *M*.*oleifera* seed + 70 g of normal feed; NFMS 10%, 10 g nonfermented *M*.*oleifera* seed + 90 g of normal feed; NFMS 20%, 20 g nonfermented *M*.*oleifera* seed + 80 g of normal feed; NFMS 30%, 30 g nonfermented *M*.*oleifera* seed + 70 g of normal feed.

The histology of the liver sections is presented in Figure 3, while that of the kidney sections is presented in Figure 4, respectively. The plate without the red arrow indicates that the central venules are normal, there is no significant observable congestion, liver cell shape (hepatocytes) is normal, and sinusoids are normal and do not contain infiltrators. Except for the mild infiltration of nuclear cytoplasm and the presence of inflammatory red cells, the liver of rats that were administered with 30% FMS showed normal hepatocytes without congestion and good architecture. Groups that were administered 20% and 30% NFMS showed mild haemorrhage across profiles. The central venules are dilated with congestion, the morphology of the hepatocytes shows the presence of inflammatory red cells, and the sinusoids appear mildly infiltrated. The treated group without a red arrow shows good kidney architecture as revealed by the normal renal cortex, glomeruli with normal mesangial cells, and capsular spaces. The renal tubules and the interstitial spaces appear normal, clear, and not congested, with well-defined profiles. Only groups that were administered with 30% FMS and NFMS showed degenerative changes, characterized by congested renal tubules (proximal and distal convoluted tubules), infiltrating renal parenchyma by red inflammatory cells, extending renal tubules, and widened capsular margins. The results of histopathology studies revealed that there is a slight impairment in the architecture of both organs. The liver of animals fed with 10% and 20% of FMS demonstrated good liver architecture characterized by normal central venules without congestion, normal hepatocytes, and sinusoids that are not infiltrated. The slight changes that were observed in animals that received 30% FMS might not point to toxicity since tested hepatic markers were not elevated in the blood. Similarly, the liver alteration might have occurred only at the specific zone of the hepatic acinus, which over time might rejuvenate by removing necrotic cells and replacing them with new ones [52, 53]. The noticeable damage observed in 20% and 30% NFMS might be responsible for the elevated GGT observed in this study. Histology from the kidney section revealed that only fortification by 30% of FMS and NFMS provoked kidney function. This trend, as similarly observed in the kidney function indices (urea and creatinine), might be attributed to the continuous elimination process of active byproducts produced from the metabolism of seed active constituents [54, 55].

## 4. Conclusion

*M. oleifera* seed is undoubtedly an excellent source of important nutrients and bioactive compounds for the maintenance of health and treatment of diseases. Investigation of the safety of FMS and NFMS in healthy albino rats at varying supplementation levels (10%, 20%, and 30%) did not significantly affect body weight or organ/body weight ratios, indicating no adverse effects on overall growth. Haematological analysis showed no detrimental effects on blood parameters, with a notable increase in PLT counts at higher supplementation levels. Biochemical assessments revealed that FMS did not alter glucose concentration, plasma protein levels, bilirubin, or liver function indices. However, higher levels (20% and 30%) of NFMS increased urea and creatinine levels, suggesting potential renal stress. Fermentation mitigated these effects by breaking down antinutritional compounds, reducing their negative impact on renal function, and making *M. oleifera* safer for dietary inclusion. Both FMS and NFMS effectively reduced lipid parameters, including cholesterol and LDL. Fermentation further enhances the bioavailability of essential nutrients such as amino acids, polyunsaturated fatty acids, vitamins, and minerals by reducing the levels of phytates, oxalates, and tannins, which can otherwise hinder nutrient absorption. This process improves digestibility and optimizes the nutritional value of *M. oleifera*, making it a better candidate for functional food applications. Oxidative stress markers indicated no significant oxidative damage to the liver and kidneys, although liver catalase activity was reduced in the NFMS group. Histological examinations revealed slight alterations in the liver and kidneys at 30% supplementation levels, more pronounced in the NFMS group. Additionally, fermentation promotes the production of beneficial microbial metabolites, supporting gut microbiota balance and improving immune function. This suggests that fermented *M. oleifera* may provide additional functional health benefits beyond basic nutrition. In conclusion, fermentation plays a crucial role in improving the safety, digestibility, and nutritional profile of *M. oleifera* seeds. By reducing antinutritional compounds, enhancing nutrient bioavailability, and minimizing potential toxicological risks, fermented *M. oleifera* demonstrates significant potential for human dietary applications. Findings from this study suggest that FMS and NFMS are safe for consumption up to 20% inclusion, with potential applications in human nutrition, functional food development, and dietary supplementation. Future studies should explore the extrapolation of these safe supplementation levels to human diets. Additionally, the potential lipid-lowering and antioxidant effects observed in this study show the role of *M. oleifera* in functional food applications, particularly for cardiovascular health and metabolic regulation.

## Figures and Tables

**Table 1:** Haematology results of albino rats that were fed with FMS and NFMS.

**Indices**	**Control**	**10%**		**20%**		**30%**	
	**Distilled H**_**2**_ **O**	**FMS**	**NFMS**	**FMS**	**NFMS**	**FMS**	**NFMS**
WBC (×10^9^/L)	10.57 ± 0.81	9.27 ± 1.38	8.80 ± 0.25	6.37 ± 1.02	13.37 ± 2.83	7.37 ± 0.66	9.57 ± 2.69
LYM (%)	74.73 ± 2.34	72.17 ± 2.46	64.67 ± 4.10^*^	72.00 ± 2.31	71.17 ± 3.72	75.03 ± 4.20	63.33 ± 2.19^*^
MID (%)	10.50 ± 0.60	10.63 ± 0.68	11.67 ± 0.88	9.60 ± 1.14	11.90 ± 0.49	10.27 ± 1.27	10.20 ± 1.33
GRAN (%)	14.77 ± 1.78	17.20 ± 2.50	23.67 ± 4.33	18.40 ± 2.30	16.93 ± 4.21	14.70 ± 5.42	26.47 ± 2.49^*^
RBC (×10^12^L)	7.16 ± 0.33	6.32 ± 1.12	5.24 ± 1.46	7.32 ± 0.13	5.79 ± 1.29	6.82 ± 0.74	6.53 ± 0.13
Hb (g/dL)	13.43 ± 0.70	12.57 ± 2.06	10.00 ± 2.63	14.10 ± 0.21	10.87 ± 2.34	13.03 ± 1.46	12.70 ± 0.32
HCT (%)	21.80 ± 7.20	36.63 ± 6.12	28.80 ± 7.31	41.67 ± 0.49^*^	32.07 ± 8.02	39.70 ± 4.46^*^	38.37 ± 0.84
PLT (×10^9^/L)	315.67 ± 4.33	374.67 ± 20.28	407.33 ± 28.42^*^	346.33 ± 21.33	435.00 ± 26.63^*^	446.33 ± 21.17^*^	430.33 ± 16.67^*^
MCV (%)	55.63 ± 0.97	58.30 ± 1.10	56.17 ± 2.07	57.50 ± 1.44	54.57 ± 2.19	58.27 ± 0.28	58.93 ± 2.51

*Note:*Value = mean ± standard error of the mean (*n* = 3‐5). Value with an asterisk (⁣^∗^) along the same column is significantly different (*p* < 0.05) relative to the control. LYM (%), MID (%), GRAN (%), RBC (×10^12^L), Hb (g/dL), HCT (%), PLT (×10^9^/L), MCV, MCH, and MCHC. FMS 10%, 10 g fermented *M*.*oleifera* seed + 90 g of normal feed; FMS 20%, 20 g fermented *M*.*oleifera* seed + 80 g of normal feed; FMS 30%, 30 g fermented *M*.*oleifera* seed + 70 g of normal feed; NFMS 10%, 10 g nonfermented *M*.*oleifera* seed + 90 g of normal feed; NFMS 20%, 20 g nonfermented *M*.*oleifera* seed + 80 g of normal feed; NFMS 30%, 30 g nonfermented *M*.*oleifera* seed + 70 g of normal feed.

Abbreviation: WBC, white blood cell.

**Table 2:** Blood chemistry of healthy rats that were fed with FMS and NFMS.

**Parameters**	**Control**	**10%**		**20%**		**30%**	
	**Distilled H**_**2**_ **O**	**FMS**	**NFMS**	**FMS**	**NFMS**	**FMS**	**NFMS**
Glu (mg/dL)	32.19 ± 0.43	31.21 ± 1.56	26.14 ± 1.28	29.90 ± 1.02	30.88 ± 5.99	28.43 ± 0.49	21.90 ± 0.86^*^
TP (g/dL)	4.21 ± 0.04	4.28 ± 0.08	4.07 ± 0.20	4.18 ± 0.15	4.22 ± 0.13	4.45 ± 0.12	4.14 ± 0.08
BIL (mmol/L)	65.19 ± 0.81	67.49 ± 0.77	65.37 ± 1.69	64.13 ± 0.53	67.49 ± 0.88	64.84 ± 0.98	73.49 ± 6.25^*^
ALT (U/L)	0.31 ± 0.01	0.29 ± 0.02	0.33 ± 0.04	0.31 ± 0.02	0.31 ± 0.02	0.31 ± 0.01	0.34 ± 0.02
AST (U/L)	123.33 ± 4.76	111.50 ± 1.26	111.50 ± 7.15	123.67 ± 3.32	131.50 ± 7.65	118.17 ± 3.49	129.50 ± 1.50
GGT (U/L)	156.90 ± 4.51	81.10 ± 5.04^*^	145.42 ± 5.13	79.75 ± 9.64^*^	193.68 ± 5.45^*^	70.61 ± 0.89^*^	195.41 ± 3.24^*^
ALP (U/L)	52.86 ± 1.56	49.57 ± 0.34	55.14 ± 3.93	50.00 ± 0.49	55.11 ± 2.03	56.71 ± 3.91	50.32 ± 1.33
UREA (mmol/L)	30.53 ± 2.77	19.43 ± 2.78	34.69 ± 5.00	21.92 ± 2.65	46.34 ± 6.01^*^	21.37 ± 2.47	52.73 ± 2.78^*^
UA (mg/dL)	2.97 ± 0.76	1.33 ± 0.21^*^	2.00 ± 0.23	2.15 ± 0.09	3.02 ± 0.36	1.28 ± 0.36^*^	3.08 ± 0.32
CREA (mg/dL)	0.51 ± 0.13	0.38 ± 0.00	1.27 ± 0.46	0.63 ± 0.13	4.20 ± 0.22^*^	0.89 ± 0.51	7.26 ± 0.58^*^
TRIG (mg/dL)	65.55 ± 10.20	80.12 ± 6.89	36.90 ± 2.12^*^	57.30 ± 4.23	79.14 ± 2.43	43.70 ± 6.57^*^	78.17 ± 5.91
CHOL (mg/dL)	158.23 ± 1.81	84.66 ± 8.73^*^	80.62 ± 4.81^*^	87.18 ± 4.48^*^	71.05 ± 1.51^*^	75.08 ± 3.53^*^	77.60 ± 5.04^*^
HDL (mg/dL)	52.16 ± 7.04	45.67 ± 7.37	52.16 ± 1.99	43.88 ± 1.18	42.76 ± 0.59	55.97 ± 2.75	50.37 ± 5.39
LDL (mg/dL)	92.95 ± 6.79	22.96 ± 1.11^*^	21.08 ± 4.44^*^	31.83 ± 3.71^*^	12.46 ± 0.50^*^	10.37 ± 3.01^*^	11.59 ± 1.24^*^

*Note:*Value = mean ± standard error of the mean (*n* = 3‐5). Value with an asterisk (⁣^∗^) along the same column is significantly different (*p* < 0.05) relative to the control. FMS 10%, 10 g fermented *M*.*oleifera* seed + 90 g of normal feed; FMS 20%, 20 g fermented *M*.*oleifera* seed + 80 g of normal feed; FMS 30%, 30 g fermented *M*.*oleifera* seed + 70 g of normal feed; NFMS 10%, 10 g nonfermented *M*.*oleifera* seed + 90 g of normal feed; NFMS 20%, 20 g nonfermented *M*.*oleifera* seed + 80 g of normal feed; NFMS 30%, 30 g nonfermented *M*.*oleifera* seed + 70 g of normal feed.

**Table 3:** Total protein and oxidative stress markers in the liver.

**Indices**	**Control**	**10%**		**20%**		**30%**	
	**Distilled H**_**2**_ **O**	**FMS**	**NFMS**	**FMS**	**NFMS**	**FMS**	**NFMS**
TP (g/L)	15.58 ± 1.4	20.39 ± 1.41	11.08 ± 1.87	24.07 ± 0.63^*^	17.10 ± 1.65	20.39 ± 3.17	15.52 ± 1.70
CAT	6.76 ± 0.46	2.20 ± 0.82^*^	2.76 ± 0.91^*^	5.18 ± 0.70	3.15 ± 0.97^*^	7.45 ± 1.60	3.27 ± 0.47^*^
SOD	0.22 ± 0.03	0.23 ± 0.01	0.18 ± 0.02	0.26 ± 0.02	0.20 ± 0.02	0.27 ± 0.00^*^	0.20 ± 0.00
GSH	1.19 ± 0.33	0.89 ± 0.18	2.68 ± 0.31^*^	0.94 ± 0.22	3.02 ± 0.61^*^	1.47 ± 0.48	4.67 ± 0.60^*^
MDA	0.23 ± 0.03	0.16 ± 0.02	0.24 ± 0.04	0.13 ± 0.01^*^	0.21 ± 0.03	0.14 ± 0.02^*^	0.16 ± 0.02^*^

*Note:*Value = mean ± standard error of the mean (*n* = 4‐5) Value with an asterisk (⁣^∗^) along the same column is significantly different (*p* < 0.05) relative to the control. FMS 10%, 10 g fermented *M*.*oleifera* seed + 90 g of normal feed; FMS 20%, 20 g fermented *M*.*oleifera* seed + 80 g of normal feed; FMS 30%, 30 g fermented *M*.*oleifera* seed + 70 g of normal feed; NFMS 10%, 10 g nonfermented *M*.*oleifera* seed + 90 g of normal feed; NFMS 20%, 20 g nonfermented *M*.*oleifera* seed + 80 g of normal feed; NFMS 30%, 30 g nonfermented *M*.*oleifera* seed + 70 g of normal feed.

Abbreviation: TP, total protein.

**Table 4:** Total protein and oxidative stress markers in the kidney.

**Indices**	**Control**	**10%**		**20%**		**30%**	
	**Distilled H**_**2**_ **O**	**FMS**	**NFMS**	**FMS**	**NFMS**	**FMS**	**NFMS**
TP (g/L)	6.52 ± 0.51	7.03 ± 1.33	4.69 ± 0.56	5.29 ± 0.30	4.81 ± 0.73	5.70 ± 0.29	6.21 ± 0.44
CAT	10.59 ± 1.23	0.49 ± 0.20^*^	4.36 ± 1.01^*^	10.11 ± 2.30	10.45 ± 1.61	13.61 ± 3.02	9.05 ± 1.41
SOD	1.26 ± 0.08	0.95 ± 0.09	1.06 ± 0.25	1.02 ± 0.03	0.72 ± 0.26^*^	1.13 ± 0.12	1.12 ± 0.22
GSH	6.35 ± 0.45	6.42 ± 1.25	4.59 ± 0.92	7.12 ± 0.14	5.31 ± 0.46	9.77 ± 1.74^*^	6.04 ± 0.34
MDA	0.45 ± 0.06	0.35 ± 0.07	0.54 ± 0.05	0.51 ± 0.04	0.54 ± 0.09	0.43 ± 0.01	0.43 ± 0.06

*Note:*Value = mean ± standard error of the mean (*n* = 4‐5) Value with an asterisk (⁣^∗^) along the same column is significantly different (*p* < 0.05) relative to the control. FMS 10%, 10 g fermented *M*.*oleifera* seed + 90 g of normal feed; FMS 20%, 20 g fermented *M*.*oleifera* seed + 80 g of normal feed; FMS 30%, 30 g fermented *M*.*oleifera* seed + 70 g of normal feed; NFMS 10%, 10 g nonfermented *M*.*oleifera* seed + 90 g of normal feed; NFMS 20%, 20 g nonfermented *M*.*oleifera* seed + 80 g of normal feed; NFMS 30%, 30 g nonfermented *M*.*oleifera* seed + 70 g of normal feed.

Abbreviation: TP, total protein.

## Data Availability

The data that support the findings of this study are available from the corresponding author upon reasonable request.
